# The association between dietary trajectories across childhood and blood pressure in early adolescence: The Longitudinal Study of Australian Children

**DOI:** 10.1038/s41430-023-01274-y

**Published:** 2023-02-16

**Authors:** Denelle Cosier, Karen Charlton, Danielle A. J. M. Schoenaker

**Affiliations:** 1 School of Medical, Indigenous and Health Sciences, University of Wollongong, Wollongong, NSW Australia; 2 Illawarra Health and Medical Research Institute, Wollongong, NSW Australia; 3 School of Primary Care, Population Sciences and Medical Education, University of Southampton, Southampton, UK; 4 NIHR Southampton Biomedical Research Centre, University of Southampton and University Hospital Southampton NHS Foundation Trust, Southampton, UK

**Keywords:** Risk factors, Epidemiology

## Abstract

**Background:**

Cardio-metabolic risk factors, including hypertension, are increasingly appearing in childhood. The aims of this study were to examine the associations between dietary trajectories across childhood and subsequent blood pressure (BP) at age 10/11, and to further determine whether these associations were explained by BMI or fat mass.

**Methods:**

Data from 4360 participants from the Longitudinal Study of Australian Children were analysed. Dietary scores were computed based on similarity of intake to the Australian Dietary Guidelines. Group-based trajectory modelling was used to identify distinct dietary trajectories based on participant’s individual dietary scores at up to four timepoints between age 4 and 11. Linear regression models examined the associations between dietary trajectories and BP measured at age 10/11. Models were adjusted for relevant covariates, and BMI or fat mass.

**Results:**

Four dietary trajectories were identified: “never healthy” (4.3%); “moderately healthy” (23.1%); “becoming less healthy” (14.2%); and “always healthy” (58.4%). Children in the “always healthy” trajectory had a lower systolic (−2.19 mmHg; 95% CI −3.78, −0.59) and diastolic BP (−1.71; −2.95, −0.47), compared with children in the “never healthy” trajectory after covariate adjustment. These associations were attenuated after additional adjustment for BMI or fat mass, but remained significant for diastolic BP.

**Conclusions:**

A dietary trajectory mostly aligned with the Australian Dietary Guidelines across childhood was associated with slightly lower BP at age 10/11, which was not fully explained by BMI or fat mass. These findings support the need to encourage and enable healthy dietary habits early in childhood to attenuate the increasing burden of cardio-metabolic disease.

## Introduction

High blood pressure (BP) is a major risk factor for adult cardiovascular disease (CVD), one of the leading causes of premature mortality globally [1]. High BP is increasingly diagnosed in children due to the rise in prevalence of children with overweight and obesity [2] and has been found to track from childhood into adulthood [3]. Without early intervention, high BP in childhood may contribute to the premature development of atherosclerosis, type 2 diabetes and CVD in adulthood [4]. Childhood also represents a critical life stage for the development of a healthy lifestyle. Among healthy lifestyle behaviours associated with CVD in adult populations, diet quality has been identified as a potential modifiable risk factor for high BP in children and adolescents [5].

Current evidence on the association of childhood diet with BP is inconclusive and contradictory. A 2018 systematic review by de Magalhães Cunha et al. [6] identified six cross-sectional studies that examined the association of dietary patterns identified using various *a posteriori* methods with BP in children and adolescents. Three of the six studies found higher BP in participants consuming a “Western” dietary pattern, and lower BP in those following healthy dietary patterns [7–9]. In contrast, one study found a lower prevalence of hypertension amongst participants with a “Modern” dietary pattern [10], and two studies found no significant associations between dietary patterns and BP [11, 12]. Since the 2018 systematic review [6], few additional cross-sectional studies have been published showing slightly lower diastolic blood pressure (DBP) [13] or systolic blood pressure (SBP) [14] among children consuming healthier dietary patterns, or no association between dietary patterns and BP [15]. Two longitudinal studies from the Netherlands found higher diet quality in early childhood (age 5 to 8) was associated with lower DBP and SBP between age 10 and 12, however, these studies have not accounted for changes in diet quality during follow-up [16, 17]. We are aware of one cohort study which used longitudinal diet data from children recruited through the Longitudinal Study of Australian Children (LSAC) who also participated in the Child Health CheckPoint at age 10/11 when detailed cardio-metabolic phenotypic outcomes were measured [18]. In this study, in which 53% of eligible children participated, a ‘never healthy’ dietary trajectory from age 2/3 to 10/11 was associated with a higher SBP (1.8 mmHg; 95% CI 0.0, 3.5) but not DBP (1.0; −0.5, 2.5) compared with the ‘always healthy’ trajectory after adjustment for age, sex, family socioeconomic position, puberty and BMI [18]. Only half of eligible children (53%) attended the Child Health CheckPoint assessments, and this study therefore likely underrepresents children from disadvantaged families who are more likely to have poor diets and at-risk vascular phenotypes [18]. Further large nationally representative longitudinal studies are needed to elucidate the association between habitual dietary intake across childhood and subsequent BP.

Despite the lack of consistent evidence from high-quality studies on the association between dietary intake and BP in children, many studies have shown that obesity is strongly associated with higher BP in this age group. A systematic review conducted by Friedemann et al. [19] found that children with overweight (*n* = 12,169 children; 8 studies) and obesity (*n* = 8074 children; 15 studies) had SBP that was 4.5 mmHg and 7.5 mmHg higher, respectively, compared to children with a normal weight. As dietary intake in childhood is strongly linked to obesity, it is important to determine if BMI and measures of body composition play a role in the association between dietary intake and BP.

The aims of this study were therefore to examine the associations between dietary trajectories across childhood and subsequent BP in early adolescence, and to further determine if these associations are explained by BMI and/or fat mass.

## Materials and methods

### Study design and population

This study used data from the Longitudinal Study of Australian Children (LSAC), which is a population-based study following the development of a nationally representative Australian sample of children and their families [20]. The LSAC commenced in 2004 and collects data every two years from two cohorts of children: the B (baby)-cohort (born March 2003–February 2004) and the K (kindergarten)-cohort (born March 1999–February 2000) [21]. Participants were sampled from the Medicare database, which is Australia’s universal healthcare programme and includes 98% of Australian children by the age of 12 months. Further details on the methodology, recruitment and response rates can be found online [21]. Families provided written informed consent to participate in the study, and the Australian Institute of Family Studies Ethics Committee approved each wave of the LSAC.

The present study includes data from waves 1 to 6 of the B-cohort and waves 1 to 4 of the K-cohort (ages 4/5 to 10/11 years), collected from 2004 to 2014 (Supplementary Table 1). A total of 5107 and 4983 children were recruited for the B- and K-cohort, of which 3764 and 4164 were retained at age 10/11 years, respectively [21]. Of the combined study sample with data at age 10/11 years (*n* = 7928), participants were excluded if parent 1 (i.e., the parent who knows the study child best and who completed the face-to-face interviews and questionnaires) was not the biological mother (*n* = 194) or if parent 2 (i.e., parent 1’s partner or another adult in the home with a parental relationship to the study child) was not the biological father (*n* = 686) to be able to account for any direct influences of parental characteristics (such as maternal hypertension in pregnancy, maternal and paternal BMI) on offspring blood pressure. Participants were also excluded if dietary intake data was not available at two or more time points (*n* = 6), if data on BP were missing at age 10/11 years (*n* = 831), or if data on relevant covariates were missing (*n* = 1851). This study therefore includes 4360 children. A comparison of characteristics of children included (*n* = 4360) and excluded (*n* = 5730) is shown in Supplementary Table 2.

### Data collection

LSAC’s data collection methods include face-to-face interviews with parents and children conducted by trained interviewers, and audio computer-assisted self-interviews completed by children.

#### Assessment of dietary intake

Dietary intake was assessed at each wave. Parents (for children aged 4-9 years) or children (from age 10) were asked a standard set of 11 questions relating to the child’s consumption of individual or grouped food or drink items [22]. Questions assessed the frequency of consumption in the previous 24 h, with answers ranging from “Not at all”, “Once” and “More than once”.

Dietary scores were derived at each wave in line with the previously developed dietary scoring system by Gasser et al. [22]. In line with the 2013 Australian Dietary Guidelines [23], partcipants were assigned an individual score (ranging from 0 to 2) for each of seven food groups: fruit, vegetables, water, fatty foods, sugary foods, sweetened drinks, and milk products or alternatives. Vegetables, fruit, water and milk products were assigned higher scores for more frequent consumption, whereas fatty foods, sugary foods and sugary drinks were assigned higher scores for less frequent consumption. The continuous scores for each food group were summed to give an overall score between 0 (least healthy) and 14 (most healthy) for each participant at each wave [22].

The dietary questions, scoring system and dietary trajectories have not been formally validated, however, the dietary intake data collected in the LSAC have previously been used in other studies, including analysis of associations with longitudinal body composition measures [24], family socioeconomic position [25], parental health behaviours [26], parenting styles [27], and preclinical cardiovascular phenotypes [18]. Moreover, the dietary trajectories derived using the scoring system by Gasser et al. [22] have been used in multiple analyses based on LSAC dietary data [18, 24–27].

#### Assessment of blood pressure

SBP and DBP measurements were taken by trained professionals in wave 6 of the B-cohort and wave 4 of the K-cohort when children were aged 10/11 using an A&D Digital Blood Pressure Monitor – Model UA-767 (A&D Co., LTD, Japan). This monitor has not been validated for use in children. Two measurements were taken by the interviewer with a one-minute interval between the measurements, according to standard protocol [28]. The mean of the two measurements was calculated and used for analysis.

#### Assessment of covariates

We identified potential covariates for the analysis based on published literature that examined the relationship between dietary intake and BP in children [7–10, 12, 29]. Questions and response options for all covariates are described in Supplementary Table 3. Variables describing age, sex, country of birth, indigenous status, socio-economic status, diabetes and hypertension in pregnancy, and maternal education were obtained from interview data collected at wave 1.The child’s birthweight was recorded by the parents at wave 1 and transformed to birthweight z-scores using the US Centres for Disease Control (CDC) growth charts [30]. Mothers were asked about breastfeeding at wave 1 (B- and K-cohort) and wave 2 (B-cohort): “Was the child ever breastfed?” (yes or no), “How old was the child when he/she had any milk or food other than breast milk (months), and “How old was the child when he/she completely stopped being breastfed (including expressed breast milk)?” (months). Based on these questions, we examined breastfeeding as never or briefly (< 1 month), 1-3 months, 4-5 months, and ≥ 6 months. Maternal and paternal weight (kg) and height (m^2^) were self-reported at wave 1 and BMI was calculated. In addition to these socio-demographic and early life factors, potential covariates at age 10/11 were considered. This included the child’s pubertal status which was categorised as “has not yet or barely started”, “definitely started”, or “seems complete” based on a series of questions asked of the child’s mother on body hair growth, voice deepening (boys only), and breast growth and first menstruation (girls only). Child’s physical activity was categorised based on questions asked of the parents on the child’s choice of how they spend their free time (active or inactive). The child’s weight was measured using Tanita body fat scales (UM-051) (Tanita Australia) [31] and height using laser stadiometers. BMI was calculated and transformed to age- and sex-adjusted BMI z-scores [32], which were then used to categorise children as underweight, normal weight, overweight or obesity according to international BMI cut-offs [33]. Percentage body fat was measured using Tanita body fat scales (UM-051) [28, 31]. The fat mass index (FMI) was calculated as fat mass (kg) divided by the square of height (m^2^), to provide a height-adjusted measures of fat mass. These anthropometric measurements were taken by trained staff.

#### Statistical analysis

Group-based trajectory modelling was used to derive trajectories based on continuous dietary scores across waves 3–6 of the B-cohort and waves 1–4 of the K-cohort (ages 4/5 to 10/11 years) separately [34]. Overall dietary scores at each wave were normally distributed and used as continuous dependent variables, and the child’s age at each wave as independent variables. For each cohort, we compared models for three, four or five trajectories, and determined if an intercept, linear, quadratic or cubic model fitted the data best. Based on Bayesian Information Criterion (BIC) values [35], four trajectories were retained in both cohorts. This is in line with the previous analysis of dietary scores and trajectories in LSAC by Gasser et al. [22].

Participant characteristics were described across the four trajectories and compared using chi-squared tests for categorical variables and one-way analysis of variance (ANOVA) for continuous variables.

Linear regression models were used to derive coefficients (non-standardised) for the association between dietary trajectories and SBP and DBP at age 10/11. To identify relevant covariates, relationships of potential covariates with dietary trajectories and SBP and DBP were examined. Chi-squared tests were used to assess differences in categorical characteristics by dietary trajectories, and ANOVA was used to examine differences in continuous characteristics by dietary trajectories. Linear regression was used to determine associations between potential covariates and SBP and DBP. Based on these preliminary analyses, covariates that were significantly associated with dietary trajectories and with SBP and/or DBP were included in the linear regression models, as well as covariates commonly used in previously published studies examining the association between diet and BP [7–10, 12, 29]. Model 1 included socio-demographic covariates (child’s age, sex, indigenous status and maternal education, country of birth and socio-economic status). Model 2 additionally adjusted for parental covariates (maternal and paternal BMI and maternal hypertension in pregnancy) and model 3 additionally adjusted for child-related covariates (breastfeeding, pubertal status and physical activity). Model 4 included all covariates included in model 3 and BMI, and model 5 included all covariates included in model 3 and FMI. Covariates that were considered but not included were: maternal diabetes in pregnancy, maternal age at delivery, child birthweight, singleton or multiple birth, sleep, and diagnosis of diabetes in the child.

A cross-product term between dietary trajectories and child sex was included in the final model to test for differences in associations between boys and girls. A cross-product term was also included between dietary trajectories and cohort to test for differences in associations between the B- and K-cohort. All statistical analyses were conducted using STATA 16.0 software [36].

## Results

**Fig. 1:**
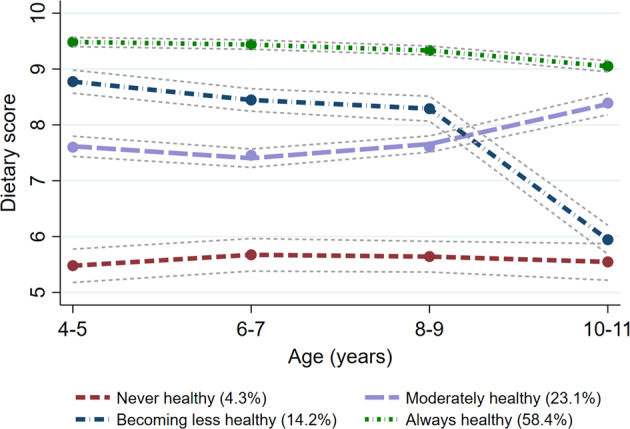
Dietary trajectories for the combined B- and K-cohorts from age 4/5 to 10/11 years, *N* = 4360. Grey dashed lines indicate 95% confidence intervals.

Dietary trajectories were computed separately for the B- and K-cohort and labelled as “always healthy”, “moderately healthy”, “becoming less healthy” and “never healthy” according to the longitudinal trends in dietary scores and in line with a previous analysis of dietary trajectories in LSAC [22]. The proportion of children in each trajectory differed between the cohorts (e.g., 46% were categorised in the “always healthy” trajectory in the B-cohort compared with 59% in the K-cohort). The overall dietary scores from age 4/5 to 10/11 also differed slightly (up to about one point out of a total of 14 points) (Supplementary Figs. 1, 2). However, patterns of changes in dietary scores within each trajectory were large comparable, and group-based trajectory modelling performed on the combined cohort was therefore used for analysis (Fig. 1).

In the combined cohort, 47.6% of children were girls, and children had a mean age of 10.4 at follow-up (SD 0.5; 62.7% were aged 10, 37.3% aged 11). Children had a mean SBP of 97.5 mmHg (SD 10.9) and mean DBP of 58.6 mmHg (SD 8.3).

Maternal, paternal and child characteristics are described in Table 1 according to dietary trajectories. Compared with children in the “never healthy” dietary trajectory, children in the “always healthy” dietary trajectory were more likely to: have a mother born overseas and with a higher educational qualification, have a mother and father with a normal BMI, be a girl, non-Indigenous, been breastfed for at least 6 months, be more physically active, and have a normal BMI (Table 1).

**Table 1:** Characteristics of children and their parents according to dietary trajectories from age 4/5 to 10/11 years, *N* = 4,360.

	Dietary trajectories				
Baseline characteristic	Never healthy	Moderately healthy	Becoming less healthy	Always healthy	*p*-value^a^
	*n* = 187 (4.3%)	*n* = 1005 (23.1%)	*n* = 621 (14.2%)	*n* = 2547 (58.4%)	
Maternal country of birth, *n* (%)					0.03
Australia, New Zealand and South Pacific Islands	171 (91.4)	863 (85.9)	516 (83.1)	2,125 (83.4)	
Europe, USA and Canada	12 (6.4)	73 (7.3)	56 (9.0)	210 (8.2)	
Asia, Middle East and Africa	4 (2.1)	69 (6.9)	49 (7.9)	212 (8.3)	
Maternal highest qualification completed, *n* (%)					<0.0001
Postgraduate degree	28 (15.0)	340 (33.8)	197 (31.7)	1,145 (45.0)	
Bachelor’s degree, diploma or certificate	68 (36.4)	334 (33.2)	218 (35.1)	827 (32.5)	
Completed high school	34 (18.2)	166 (16.5)	107 (17.2)	325 (12.8)	
Did not complete high school	57 (30.5)	165 (16.4)	99 (15.9)	57 (30.5)	
Maternal age at birth of study child, *n* (%)					0.13
< 25 years	24 (12.8)	102 (10.2)	64 (10.3)	205 (8.1)	
> 25 < 35 years	124 (66.3)	684 (68.1)	433 (69.7)	1,781 (69.9)	
> 35 years	39 (20.9)	219 (21.8)	124 (20.0)	561 (22.0)	
Maternal hypertension in pregnancy of study child, *n* (%)	17 (9.1)	68 (6.8)	44 (7.1)	200 (7.9)	0.56
Maternal diabetes in pregnancy of study child, *n* (%)	12 (6.5)	45 (4.5)	36 (5.8)	137 (5.4)	0.54
Maternal BMI, *n* (%)					<0.0001
< 25 kg/m^b^	72 (38.5)	559 (55.6)	331 (53.3)	1,546 (60.7)	
25–29.9 kg/m^b^	54 (28.9)	270 (26.9)	168 (27.1)	636 (25.0)	
≥ 30 kg/m^b^	61 (32.6)	176 (17.5)	122 (19.7)	365 (14.3)	
Paternal BMI, *n* (%)					0.01
< 25 kg/m^b^	35 (18.7)	268 (26.7)	160 (25.8)	756 (29.7)	
25–29.9 kg/m^b^	78 (41.7)	433 (43.1)	276 (44.4)	1,096 (43.0)	
≥ 30 kg/m^b^	46 (24.6)	168 (16.7)	102 (16.4)	380 (14.9)	
Missing BMI data	28 (15.0)	136 (13.5)	83 (13.4)	315 (12.4)	
Child sex, *n* (% girls)	70 (37.4)	477 (47.5)	279 (44.9)	1,251 (49.1)	0.01
Child indigenous status, *n* (% indigenous)	7 (3.7)	24 (2.4)	16 (2.6)	34 (1.3)	0.01
Child birthweight z-score, mean (SD)	-0.03 (1.05)	0.07 (1.03)	0.08 (1.05)	0.01 (1.06)	0.21
Child breastfeeding, *n* (%)					<0.0001
Any breastfeeding ≥ 6 months	64 (34.2)	547 (54.4)	338 (54.4)	1,594 (62.6)	
Any breastfeeding ≥ 3 months–< 6 months	44 (23.5)	193 (19.2)	128 (20.6)	444 (17.4)	
Any breastfeeding ≥ 1 month–< 3 months	21 (11.2)	83 (8.3)	49 (7.9)	205 (8.1)	
No or any breastfeeding < 1 month	58 (31.0)	182 (18.1)	106 (17.1)	304 (11.9)	
Child age at follow-up, mean (SD)^b^	10.4 (0.5)	10.4 (0.5)	10.3 (0.5)	10.4 (0.5)	0.30
Child SEIFA relative disadvantage at follow-up, *n* (%)^b^					<0.0001
Quintile 1 (most disadvantaged)	38 (20.3)	168 (16.7)	92 (14.8)	309 (12.1)	
Quintile 2	31 (16.6)	196 (19.5)	123 (19.8)	416 (16.3)	
Quintile 3	50 (26.7)	219 (21.8)	126 (20.3)	521 (20.5)	
Quintile 4	36 (19.3)	216 (21.5)	140 (22.5)	518 (20.3)	
Quintile 5 (least disadvantaged)	32 (17.1)	206 (20.5)	140 (22.5)	783 (30.7)	
Child pubertal status at follow-up, *n* (%)^b^					0.82
Has not yet or barely started	137 (73.3)	719 (71.5)	442 (71.2)	1,804 (70.8)	
Has definitely started	43 (23.0)	253 (25.2)	165 (26.6)	663 (26.0)	
Seems complete	7 (3.7)	33 (3.3)	14 (2.3)	80 (3.1)	
Child physical activity at follow-up, *n* (%)^b^					0.01
Usually chooses inactive	78 (41.7)	340 (33.8)	207 (33.3)	819 (32.2)	
Usually chooses active	69 (36.9)	426 (42.4)	294 (47.3)	1,087 (42.7)	
Equally likely to choose active or inactive	40 (21.4)	239 (23.8)	120 (19.3)	641 (25.2)	
Child BMI at follow-up (kg/m^b^), mean (SD)^b^	20.1 (4.1)	19.2 (3.4)	18.9 (3.3)	18.7 (3.1)	<0.0001
Child BMI category^c^ at follow-up, *n* (%)^b^					<0.0001
Underweight	6 (3.2)	36 (3.6)	21 (3.4)	96 (3.8)	
Normal weight	106 (56.7)	651 (64.8)	420 (67.6)	1,815 (71.3)	
Overweight	45 (24.1)	235 (23.4)	141 (22.7)	509 (20.0)	
Obesity	30 (16.0)	83 (8.3)	39 (6.3)	127 (5.0)	
Child fat mass index at follow-up, mean (SD)^b^	5.2 (2.9)	4.6 (2.4)	4.4 (2.2)	4.3 (2.1)	<0.0001
Systolic blood pressure at follow-up, mean (SD)^b^	100.5 (11.3)	98.3 (10.8)	97.4 (10.1)	97.0 (11.1)	<0.0001
Diastolic blood pressure at follow-up, mean (SD)^b^	60.5 (7.8)	59.4 (7.9)	58.6 (8.2)	58.2 (7.4)	<0.0001

*BMI* Body mass index, *SEIFA* Socio-Economic Indexes for Areas.

^a^*p*-value from chi-square test or ANOVA.

^b^Follow-up at time of blood pressure measurement: wave 6 of B-cohort or wave 4 of K-cohort.

^c^Age- and sex-adjusted BMI z-scores were used to categorise children according to international BMI cut-offs [33].

Associations between dietary trajectories and SBP and DBP are shown in Table 2. The “becoming less healthy” and “always healthy” dietary trajectories were associated with lower SBP and DBP compared with the “never healthy” trajectory after adjustment for child and maternal socio-demographic covariates (model 1). These results were not substantially influenced by further adjustments for parental (model 2) and child-related covariates (model 3). After adjustment for socio-demographic factors and parental and child-related characteristics (model 3), children in the “becoming less healthy” and “always healthy” dietary trajectories had lower SBP (−2.19 mmHg; 95% CI −3.93, −0.45 and −2.19; −3.78, −0.59, respectively) and DBP (−1.54; −2.89, −0.20 and −1.71; −2.95, −0.47, respectively) at age 10/11 compared with children in the “never healthy” dietary trajectory. These associations were attenuated after adjustment for BMI (model 4) and FMI (model 5) but remained significant for the “always healthy” dietary trajectory compared with the “never healthy” dietary trajectory in relation with DBP (−1.34; −2.54, −0.14 after adjustment for BMI, and −1.23; −2.43, −0.03 after adjustment for FMI). The “moderately healthy” dietary trajectory was not associated with SBP nor DBP.

**Table 2:** Regression coefficients for associations between dietary trajectories and systolic and diastolic blood pressure at age 10/11 years, *N* = 4360.

	Dietary trajectories			
	Never healthy *n* = 187 (4.3%)	Moderately healthy *n* = 1005 (23.1%)	Becoming less healthy *n* = 621 (14.2%)	Always healthy *n* = 2547 (58.4%)
	Coefficient (95% CI)	Coefficient (95% CI)	Coefficient (95% CI)	Coefficient (95% CI)
Systolic blood pressure, mmHg				
Model 1^a^	Reference	−1.98 (−3.67, −0.28)	−2.86 (−4.64, −1.08)	−3.03 (−4.66, −1.40)
Model 2^b^	Reference	−1.29 (−2.97, 0.38)	−2.27 (−4.03, −0.52)	−2.26 (−3.88, −0.65)
Model 3^c^	Reference	−1.21 (−2.87, 0.46)	−2.19 (−3.93, −0.45)	−2.19 (−3.78, −0.59)
Model 4^d^	Reference	−0.83 (−2.34, 0.67)	−1.28 (−2.85, 0.29)	−1.37 (−2.82, 0.07)
Model 5^e^	Reference	−0.70 (−2.22, 0.82)	−1.21 (−2.80, 0.38)	−1.17 (−2.64, 0.29)
Diastolic blood pressure, mmHg				
Model 1^a^	Reference	−1.16 (−2.46, 0.13)	−1.95 (−3.30, −0.60)	−2.24 (−3.48, −1.00)
Model 2^b^	Reference	−0.83 (−2.12, 0.46)	−1.66 (−3.01, −0.32)	−1.88 (−3.12, −0.64)
Model 3^c^	Reference	−0.70 (−1.98, 0.59)	−1.54 (−2.89, −0.20)	−1.71 (−2.95, −0.47)
Model 4^d^	Reference	−0.53 (−1.77, 0.71)	−1.13 (−2.43, 0.18)	−1.34 (−2.54, −0.14)
Model 5^e^	Reference	−0.46 (−1.70, 0.79)	−1.08 (−2.38, 0.23)	−1.23 (−2.43, −0.03)

^a^Model 1 adjusted for socio-demographic covariates: child’s age, sex, indigenous status, socio-economic status; maternal education and country of birth.

^b^Model 2 adjusted for covariates in model 1 + for parental covariates: maternal and paternal body mass index and maternal hypertension in pregnancy.

^c^Model 3 adjusted for covariates in model 2 + child-related covariates: breastfeeding, pubertal status and physical activity.

^d^Model 4 adjusted for covariates in model 3 + child body mass index.

^e^Model 5 adjusted for covariates in model 3 + child fat mass index.

In addition, p-values for interaction indicated no differences in associations between dietary trajectories and BP by child sex (p-value for interaction 0.62 and 0.54 for SBP and 0.54 and 0.56 for DBP, for cross-product terms in model 4 and 5 respectively) or by cohort (0.28 and 0.20 for SBP and 0.65 and 0.64 for DBP).

## Discussion

In this population-based longitudinal cohort study, we observed that children who consistently had the highest dietary scores from age 4/5 to 10/11 (“always healthy” trajectory; 58%) and whose dietary scores were high but decreased from age 8/9 (“becoming less healthy” trajectory; 14%) had a slightly lower SBP and DBP at age 10/11 compared with children who consistently had the lowest dietary scores (“never healthy” trajectory; 4%), after adjustment for child and parental characteristics. These associations were attenuated after additional adjustment for child BMI or fat mass, except for the association between the “always healthy” dietary trajectory and lower DBP.

These findings provide an important contribution to the current evidence on childhood diet quality and BP, which is based on a limited number of cross-sectional [6, 13, 14] and longitudinal studies [16–18]. In line with our findings, few previous studies have observed higher BP among children consuming generally less healthy diets. Two cross-sectional studies among 12–19 year old Iranian adolescents (*n* = 557) [7] and among 14–18 year old US adolescents (*n* = 649) [14] found higher SBP (3.16 mmHg; 95% CI 1.09, 5.22 and 6.9; 2.5, 11.4, respectively) among children consuming “unhealthy” compared with “healthy” dietary patterns. Two other studies, among Australian adolescents (*n* = 764, 12–18 years) [9] and among Spanish children (*n* = 793, 5–16 years) [13], found higher DBP (2.2 mmHg and 1 mmHg; *p* < 0.05, respectively) among children with lower scores on a “Fruit, salad, cereal and fish” dietary pattern or when comparing a “Processed” with “Health Conscious” dietary pattern. In a Dutch cohort, a one-unit higher diet quality score at age 8 was associated with lower SBP (−0.04 SD; 95% CI −0.06, −0.01) and DBP (−0.05; −0.07, −0.02) at age 10 [17]. When considering longitudinal diet data among 1,861 children who participated in the LSAC and the Child Health Checkpoint, the “never healthy” dietary trajectory was associated with a higher SBP (1.8 mmHg; 95% CI 0.0, 3.5) but not DBP (1.0; −0.5, 2.5) compared with the “always healthy” trajectory [18]. Findings from these studies were all adjusted for child BMI. In a study among Chinese children (*n* = 5267, mean age 9.5) [8], a “Western” dietary pattern was associated with a higher SBP (6.2 mmHg) and DBP (5.0 mmHg) (both *p* < 0.05) compared with a “Healthy” dietary pattern, however, these stronger associations were adjusted only for age and sex. In line with findings from Kerr etal. [18], our findings on longitudinal dietary trajectories demonstrate the importance of changes in diet quality across childhood. In addition to the potential benefit of the “always healthy” trajectory on BP, our findings suggest that relatively higher diet quality in early childhood, which becomes worse from age 8/9 (“becoming less healthy” trajectory) may not have a negative impact on BP at age 10/11, although further follow-up is required to determine if worsening diet quality influences later BP. Our findings suggested no differences in associations between dietary trajectories and BP by sex and cohort. Limited number of previous studies that have examined sex differences are inconclusive [17, 18]. Collectively, current evidence suggests that BP may be slightly higher among children consuming less healthy diets. Findings from our study add to this evidence by suggesting that this association may in part, but not fully, be explained by BMI and fat mass. Further studies that track diet quality and BP across childhood and into adulthood are required to determine the influences of changes in diet quality over time.

The 1 to 2 mmHg difference in SBP and DBP observed in our study and previous studies comparing children with more healthy and less healthy diets is clinically relevant at the population level. A large study among adults in the US has shown that a 1 mmHg decrease in SBP results in 9.0 and 13.5 fewer coronary heart disease, 4.8 and 12.1 fewer stroke and 13.3 and 20.3 fewer heart failure events per 100,000 person-years for White and African-American people, respectively [37]. Similar to the relationship between BP and CVD in adults, it has been hypothesised that the association between BP and subclinical atherosclerosis and target organ damage in children is linear, and that even small increases in SBP and DBP are associated with hypertension in adulthood [5]. Moreover, the difference in BP already observed in childhood may become larger over time, especially as poor dietary habits and elevated BP levels in childhood have been shown to track into adulthood [5]. It is therefore essential to encourage and enable the consumption of healthy diets in line with guidelines from an early age to prevent any elevations in BP and the subsequent increased risk of developing cardio-metabolic disease in later life.

The majority of children in our study were categorised in the “always healthy” dietary trajectory (58%), however, their diet quality was still sub-optimal and did not reach the maximum dietary score. This is in line with the concerning finding that, based on the latest national nutrition survey data, almost all (99%) Australian children aged 2 to 18 do not meet the recommended daily serves of vegetables, and 30% of their total energy intake comes from discretionary foods [38]. Interventions to prevent and improve the poor diet quality of children should address its inter-related determinants, including children’s physical and social environments [39]. Such interventions have the potential to minimise increases in BP from childhood through to adolescence and early adulthood, and lay the foundation for lifelong healthy dietary habits.

Strengths of our study include the large national sample of children, the regular long-term follow-up, the objective measurements of child height, weight, fat mass and BP, and the adjustment for a range of child, maternal and paternal covariates. To our knowledge, our study is the largest and most representative longitudinal study examining associations of dietary trajectories across childhood with BP at age 10/11. The study also has limitations. The dietary questions, scoring system and dietary trajectories used in the LSAC have not been previously validated, only reflect frequency of consumption in the previous 24 h, and are not detailed enough to quantify the intake of specific nutrients and foods. This prevents a comparison of children’s dietary intake across dietary trajectories and comparison to recommended reference standards due to lack of inclusion of all food groups listed in the Australian Dietary Guidelines [23] (i.e. cereals and meat/meat alternatives), as well as adjustment for total energy intake as a potential covariate. Moreover, assessment of covariates was based on self-reported data and simplified non-validated questions, for example for physical activity and pubertal status, which may have influenced accurate covariate adjustment. Further, the BP device used in the LSAC was not validated for use in children. This is an important limitation and may have biased our findings. Any bias or misclassification is however likely to be non-differential as the same model was used to measure BP among all children in LSAC using a standard protocol. In addition, mean arterial pressure (MAP, potentially a better indicator of perfusion to vital organs than SBP) was not measured and may be explored in future studies. While Tanita weighing scales, which use Bioelectrical impedance analysis (BIA) to estimate percentage body fat, may not have provided the most accurate estimate as it may have been influenced, for example, by differences in children’s hydration status, it is a common and appropriate method for use in large studies among children because of its low cost and ease of use [40]. Also, we excluded 57% of children who were lost-to-follow up or had missing data. This is a common problem in large and long-running cohort studies in which detailed measurements are taken such as weight and BP. A comparison of key characteristics between participants who were included and excluded showed small differences at age 10/11 in dietary scores (mean score 8.2 vs 8.1), SBP (97.5 vs 99.2 mmHg) and DBP (58.6 vs 59.9 mmHg), respectively. Participants who were excluded were, in general, more likely to report poorer child and parental health and health behaviours. This suggests that the association between dietary trajectories and BP may have been underestimated.

## Conclusion

Our findings suggest that children with a dietary trajectory consistently in line with the Australian Dietary Guidelines throughout childhood have a slightly lower DBP in early adolescence compared to children with a less healthy diet. As dietary habits established in childhood are likely to continue into adulthood, it is essential to encourage and enable healthy dietary intake early in childhood to prevent the development of cardio-metabolic disease across the life course.

## Supplementary information

Supplementary Information

## Data Availability

Data are available from Growing Up in Australia for researchers who meet the criteria for access to confidential data, and can be requested at https://growingupinaustralia.gov.au/data-and-documentation/accessing-lsac-data.
