# Supplementary material for: The association between dietary trajectories across childhood and blood pressure in early adolescence: The Longitudinal Study of Australian Children
Source: Eur J Clin Nutr. 2023 Feb 16;77(6):677–83. doi: 10.1038/s41430-023-01274-y (PMC10247355; doi:10.1038/s41430-023-01274-y)
Supplement: Supplementary file 1 — Supplementary Information [file 41430_2023_1274_MOESM1_ESM.docx]

**Supplementary information**

**Supplementary Figure 1.** Dietary trajectories for the B-cohort from age 4/5 to 10/11 years, N = 2,383.

**Supplementary Figure 2.** Dietary trajectories for the K-cohort from age 4/5 to 10/11 years, N = 1,977.

**Supplementary Table 1.** Overview of longitudinal data collected in the B- and K-cohorts of the Longitudinal Study of Australian Children and used in analyses examining associations between dietary trajectories and blood pressure.

**Supplementary Table 2.** Comparison of characteristics between participants included and excluded for the current analysis

**Supplementary Table 3.** Description of covariates used in analyses examining associations between dietary trajectories and blood pressure


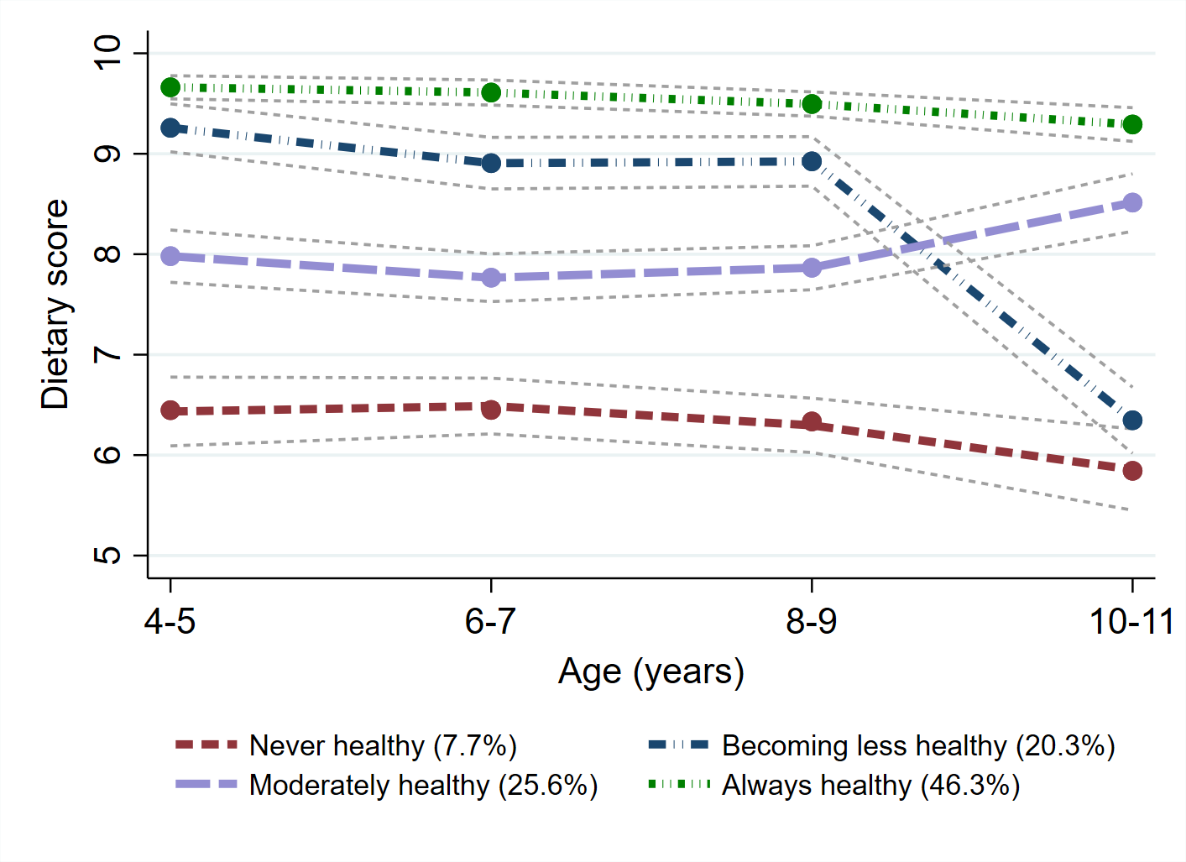


**Supplementary Figure 1.** Dietary trajectories for the B-cohort from age 4/5 to 10/11 years, N = 2,383. Grey dashed lines indicate 95% confidence intervals.


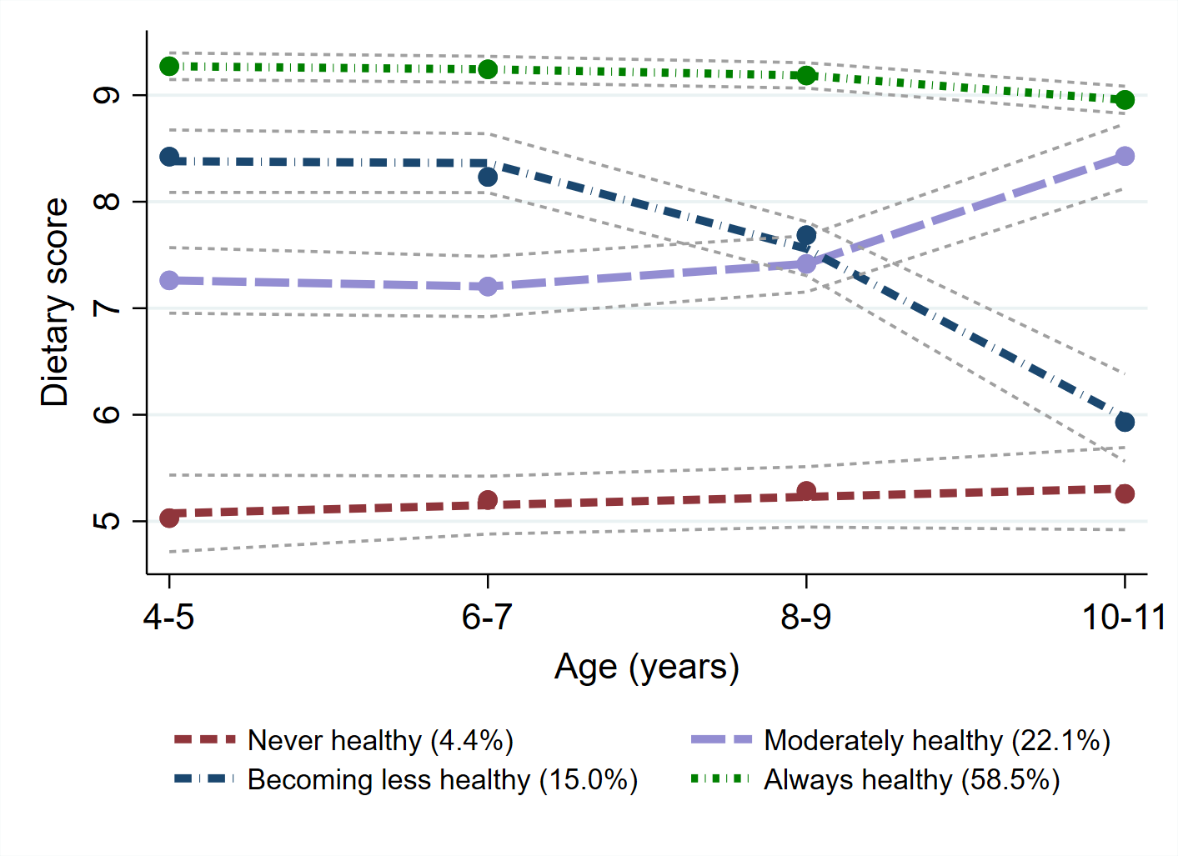


**Supplementary Figure 2.** Dietary trajectories for the K-cohort from age 4/5 to 10/11 years, N = 1,977. Grey dashed lines indicate 95% confidence intervals.

**Supplementary Table 1.** Overview of longitudinal data collected in the B- and K-cohorts of the Longitudinal Study of Australian Children and used in analyses examining associations between dietary trajectories and blood pressure.

| **B-cohort** | **Wave 1** | **Wave 2** | **Wave 3** | **Wave 4** | **Wave 5** | **Wave 6** |
| --- | --- | --- | --- | --- | --- | --- |
| Year | 2004 | 2006 | 2008 | 2010 | 2012 | 2014 |
| Age (years) | 0-1 | 2-3 | 4-5 | 6-7 | 8-9 | 10-11 |
|  |  |  |  |  |  |  |
| **K-cohort** |  |  | **Wave 1** | **Wave 2** | **Wave 3** | **Wave 4** |
| Year |  |  | 2004 | 2006 | 2008 | 2010 |
| Age (years) |  |  | 4-5 | 6-7 | 8-9 | 10-11 |
|  |  |  |  |  |  |  |
| **Study child characteristics** |  |  |  |  |  |  |
| Dietary intake |  |  | BK | BK | BK | BK |
| Blood pressure |  |  |  |  |  | BK |
| Age |  |  |  |  |  | BK |
| Sex | B |  | K |  |  |  |
| BMI |  |  |  |  |  | BK |
| Fat-mass index |  |  |  |  |  | BK |
| Physical activity |  |  |  |  |  | BK |
| Pubertal status |  |  |  |  |  | BK |
| Socioeconomic status |  |  |  |  |  | BK |
| Indigenous status | B |  | K |  |  |  |
| Birthweight | B |  | K |  |  |  |
| Breastfeeding | B | B | K |  |  |  |
|  |  |  |  |  |  |  |
| **Parental characteristics** |  |  |  |  |  |  |
| Maternal education level | B |  | K |  |  |  |
| Maternal country of birth | B |  | K |  |  |  |
| Gestational diabetes | B |  | K |  |  |  |
| Gestational hypertension | B |  | K |  |  |  |
| Maternal age at birth of study child | B |  | K |  |  |  |
| Maternal BMI | B |  | K |  |  |  |
| Paternal BMI | B |  | K |  |  |  |

**Supplementary Table 2.** Comparison of characteristics between participants included and excluded for the current analysis

| Characteristic | Included  n = 4,360 | Excluded  n = 5,730 | p-value^1^ |
| --- | --- | --- | --- |
| Maternal country of birth, n (%) |  |  | <0.0001 |
| Australia, New Zealand and South Pacific Islands | 3,675 (84.3) | 4,354 (80.2) |  |
| Europe, USA and Canada | 351 (8.1) | 367 (6.8) |  |
| Asia, Middle East and Africa | 334 (7.7) | 710 (13.1) |  |
| Maternal highest qualification completed, n (%) |  |  | <0.0001 |
| Postgraduate degree | 1,710 (39.2) | 1,368 (23.9) |  |
| Bachelor’s degree, diploma or certificate | 1,447 (33.2) | 2,143 (37.5) |  |
| Completed high school | 632 (14.5) | 877 (15.3) |  |
| Did not complete high school | 571 (13.1) | 1,334 (23.3) |  |
| Maternal hypertension in pregnancy of study child, n (%) | 329 (7.6) | 321 (8.1) | 0.32 |
| Maternal BMI, n (%) |  |  | 0.11 |
| <25 kg/m^2^ | 2,508 (57.5) | 1,996 (55.7) |  |
| 25-29.9 kg/m^2^ | 1,128 (25.9) | 931 (26.0) |  |
| ≥30 kg/m^2^ | 724 (16.6) | 656 (18.3) |  |
| Paternal BMI, n (%) |  |  | <0.0001 |
| <25 kg/m^2^ | 1,219 (28.0) | 985 (17.2) |  |
| 25-29.9 kg/m^2^ | 1,883 (43.2) | 1,205 (21.0) |  |
| ≥30 kg/m^2^ | 696 (16.0) | 498 (8.7) |  |
| Missing BMI data | 562 (12.9) | 3,042 (53.1) |  |
| Child sex, n (% girls) | 2,077 (47.6) | 2,869 (50.1) | 0.02 |
| Child indigenous status, n (% indigenous) | 81 (1.9) | 336 (5.9) | <0.0001 |
| Child breastfeeding, n (%) |  |  | <0.0001 |
| Any breastfeeding ≥6 months | 2,543 (58.3) | 2,751 (48.1) |  |
| Any breastfeeding ≥3 months - <6 months | 809 (18.6) | 966 (16.9) |  |
| Any breastfeeding ≥1 month - <3 months | 358 (8.2) | 636 (11.1) |  |
| No or any breastfeeding <1 month | 650 (14.9) | 1,366 (23.9) |  |
| Child age at follow-up, mean (SD)^2^ | 10.4 (0.5) | 10.4 (0.5) | 0.69 |
| Child SEIFA relative disadvantage at follow-up, n (%)^2^ |  |  | <0.0001 |
| Quintile 1 (most disadvantaged) | 607 (13.9) | 777 (21.8) |  |
| Quintile 2 | 766 (17.6) | 701 (19.6) |  |
| Quintile 3 | 916 (21.0) | 747 (20.9) |  |
| Quintile 4 | 910 (20.9) | 664 (18.6) |  |
| Quintile 5 (least disadvantaged) | 1,161 (26.6) | 683 (19.1) |  |
| Child pubertal status at follow-up, n (%)^2^ |  |  | 0.04 |
| Has not yet or barely started | 3,102 (71.2) | 2,313 (68.9) |  |
| Has definitely started | 1,124 (25.8) | 914 (27.2) |  |
| Seems complete | 134 (3.1) | 131 (3.9) |  |
| Child physical activity at follow-up, n (%)^2^ |  |  | <0.0001 |
| Usually chooses inactive | 1,444 (33.1) | 1,199 (32.8) |  |
| Usually chooses active | 1,876 (43.0) | 1,446 (39.6) |  |
| Equally likely to choose active or inactive | 1,040 (23.9) | 1,009 (27.6) |  |
| Child dietary score at follow-up, mean (SD)^2^ | 8.2 (2.0) | 8.1 (2.1) | <0.0001 |
| Child systolic blood pressure at follow-up, mean (SD)^2^ | 97.5 (10.9) | 99.2 (11.6) | <0.0001 |
| Child diastolic blood pressure at follow-up, mean (SD)^2^ | 58.6 (8.3) | 59.9 (8.7) | <0.0001 |
| Child BMI at follow-up (kg/m^2^), mean (SD)^2^ | 18.9 (3.2) | 18.9 (4.3) | <0.0001 |
| Child BMI category^3^ at follow-up, n (%)^2^ |  |  | <0.0001 |
| Underweight | 159 (3.7) | 216 (6.7) |  |
| Normal weight | 2,992 (68.6) | 2,063 (64.0) |  |
| Overweight | 930 (21.3) | 667 (20.7) |  |
| Obesity | 279 (6.4) | 279 (8.7) |  |
| Child fat mass index at follow-up, mean (SD)^2^ | 4.4 (2.2) | 4.6 (2.6) | <0.0001 |

BMI, body mass index; SEIFA, Socio-Economic Indexes for Areas.

^1^ p-value from chi-square or t-test.

^2^ follow-up at time of blood pressure measurement: wave 6 of B-cohort or wave 4 of K-cohort.

^3^ age- and sex-adjusted BMI z-scores were used to categorise children according to international BMI cut-offs

**Supplementary Table 3.** Description of covariates used in analyses examining associations between dietary trajectories and blood pressure

| **Confounder** | **Measure and additional information** |
| --- | --- |
| Physical activity | During the face-to-face interviews, parents were asked “What does child usually do when she/he has a choice about how to spend free time?” (1).  The options for answering were:   - Usually chooses inactive free time like television, computer, drawing or reading - Just as likely to choose active as inactive past-time - Usually chooses active past-time like bike riding, dancing, games or sport. |
| Sex | Categorised as male or female. |
| Child’s Age | Based on the child’s date of birth and date of study completion (1). |
| Pubertal Status | Pubertal characteristics of the study child were reported from age 8-9years onwards, through interviews with the child’s mother. Mothers were asked about body hair growth, skin changes, voice deepening (male child only), facial hair (male child only) and breast growth (female child only) (1). Options for answering were: *Has not yet started; has barely started; has definitely started; seems complete.*  For this study, answers were categorised as:   - Has not yet or barely started - Has definitely started - Seems complete |
| Socioeconomic status | This was measured through the Socio-economic indexes for areas (SEIFA) index of relative disadvantage. This measure was obtained from the Australian Bureau of Statistics and was linked to Statistical Local Area’s (SLA) in Australia (2). The index of relative advantage was divided into deciles, with the higher scores having the lowest rating of disadvantage. An SLA had a high score if there were few households with low income, few people with no qualifications or few people with low-skilled qualifications (2). We divided the SEIFA index into quintiles from least disadvantaged to most disadvantaged. |
| Maternal education level | This was measured at the first face-to-face interview by asking the study child’s mother a series of questions related to their highest level of education:  “What was the highest year of primary or secondary school Mother completed?” *Year 12 or equivalent; Year 11 or equivalent; Year 10 or equivalent; Year 9 or equivalent; Year 8 or below; Never attended school; Still at school*  “Has Mother completed a trade certificate or any other educational qualification?” *No; No, still studying for first qualification; Yes, trade certificate/apprenticeship; Yes, other qualification*  “Has Mother completed a trade certificate or any other educational qualification?” *Yes; No*  “What is the level of the highest qualification that Mother completed?” *Postgraduate degree; Graduate diploma/certificate; Bachelor degree; Advanced diploma/diploma; Certificate; Other* (1).  For this study, data were transformed to categorise answers as:   - Postgraduate degree - Bachelor’s degree, diploma or certificate - Completed high school - Did not complete high school |
| Maternal hypertension in pregnancy | The mother was asked “During this pregnancy, did you have high blood pressure needing treatment (admission to hospital or medication)?” with the answer either being “yes” or “no” (1). |
| Maternal country of birth | The mother was asked what country they were born in during the first face-to-face interview (1). The answers were categorised according to the Standard Australian Classification of Countries (3). |
| Child indigenous status | The mother was asked “Is Study Child of Aboriginal or Torres Strait Islander origin?” (1)  The answers were dichotomised as being “yes” for children who were of Aboriginal and/or Torres Strait Islander and “no” for those who weren’t. |
| Maternal BMI | Maternal BMI was calculated as weight (kg) divided by height squared (m^2^) using self-reported measurements obtained at the first face-to-face interview. Parental BMI was categorised as underweight (<18.5kg/m^2^), normal weight (18.5-24.9 kg/m^2^), overweight (25-29.9 kg/m^2^) or obese (>30 kg/m^2^) according to the World Health Organisation BMI classification (4). |
| Paternal BMI | See maternal BMI. |
| Breastfeeding | Parents at the first face-to-face interview were asked a series of questions relating to study child’s breastfeeding status:  “Was child ever breastfed?” *Yes; No*  “Is child still being breastfed?” *Yes; No*  “How old was child when he/she completely stopped being breastfed?” *Number (age in days); -1 (still having breast milk)* (1).  For this study, data were transformed to categorise breastfeeding as:   - Any breastfeeding ≥6 months - Any breastfeeding ≥3 months - <6 months - Any breastfeeding ≥1 month - <3 months - No or any breastfeeding <1 month |

**References:**

1. Growing Up in Australia. Data and Documentation: Australian Institute of Family Studies 2020 [Available from: <https://growingupinaustralia.gov.au/data-and-documentation/data-dictionary/downloads>.

2. Australian Bureau of Statistics. 2033.0.55.001 - Census of Population and Housing: Socio-Economic Indexes for Areas (SEIFA), Australia, 2016 Canberra ACT,: Australian Bureau of Statistics,; 2018 [Available from: <https://www.abs.gov.au/ausstats/abs@.nsf/mf/2033.0.55.001>.

3. Australian Bureau of Statistics. 1269.0 - Standard Australian Classification of Countries (SACC), Second Edition Canberra ACT,: Australian Bureau of Statistics,; 2008 [Available from: <https://www.abs.gov.au/ausstats/abs@.nsf/0/C8B8914F6C683351CA25744D00818CED?opendocument>.

4. World Health Organisation. Body mass index - BMI: WHO; 2020 [Available from: <http://www.euro.who.int/en/health-topics/disease-prevention/nutrition/a-healthy-lifestyle/body-mass-index-bmi>.
